# Liquid Metal‐Vitrimer Conductive Composite for Recyclable and Resilient Electronics

**DOI:** 10.1002/adma.202501341

**Published:** 2025-06-01

**Authors:** Dong Hae Ho, Meng Jiang, Ravi Tutika, Joshua C. Worch, Michael D. Bartlett

**Affiliations:** ^1^ Mechanical Engineering Soft Materials and Structures Lab Virginia Tech Blacksburg VA 24061 USA; ^2^ Macromolecules Innovation Institute Virginia Tech Blacksburg VA 24061 USA; ^3^ Department of Chemistry Worch Lab Blacksburg VA 24061 USA

**Keywords:** composite, electrical conductivity, liquid metal, recyclable electronics, vitrimer

## Abstract

Electronic devices are ubiquitous in modern society, yet their poor recycling rates contribute to substantial economic losses and worsening environmental impacts from electronic waste (E‐waste) disposal. Here, recyclable and healable electronics are reported through a vitrimer‐liquid metal (LM) microdroplet composite. These electrically conductive, yet plastic‐like composites display mechanical qualities of rigid thermosets and recyclability through a dynamic covalent polymer network. The composite exhibits a high glass transition temperature, good solvent resistance, high electrical conductivity, and recyclability. The vitrimer synthesis proceeds without the need for a catalyst or a high curing temperature, which enables facile fabrication of the composite materials. The as‐synthesized vitrimer exhibits a fast relaxation time with reconfigurability and shape memory. The electrically conductive composite exhibits high electrical conductivity with LM volume loading as low as 5 vol.%. This enables the fabrication of fully vitrimer‐based circuit boards consisting of sensors and indicator LEDs integrated with LM‐vitrimer conductive wiring. Electrical self‐healing and thermally triggered material healing are further demonstrated with the composites. The vitrimer and LM‐composite provide a pathway toward fully recyclable, mechanically robust, and reconfigurable electronics, thus advancing the field of electronic materials.

## Graphical abstract

To address the growing electronic waste challenge, an electrically conductive, healable, and recyclable plastic composite is created, composed of vitrimer and liquid metal microdroplets. A reconfigurable circuit board is demonstrated using this composite material, providing a notable step toward sustainable and robust electronic technologies.

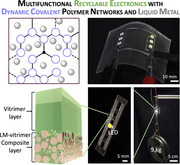

## 1 Introduction

The rapid development and expanded use of electronic devices in the modern era has exacerbated the growing issue of electronic waste (E‐waste).^[^ ^1^ ^]^ Some components of E‐waste, such as gold electrodes and other precious metals, can be partially recovered by chemical treatment processes involving strong acids and later repurposed into new electronics parts.^[^ ^2^ ^]^ However, many modern electronic devices are high‐performance composites featuring non‐recyclable thermosetting plastics, such as epoxy‐laminated fiberglass sheets, as a base material. The multi‐component nature of E‐waste complicates separation and/or recycling efforts, especially due to the extreme durability and chemical resistance of thermosets. As such, efforts to address the recycling inefficiencies of E‐waste materials have been aimed at improving the recyclability or processability of the polymeric components.

The rise of organic semiconducting polymers in the 1990's–2000's signaled a path to flexible conductive plastics.^[^ ^3^ ^]^ However, these materials have challenges related to ambient stability^[^ ^4^ ^]^ and are typically brittle primarily due to their low molecular weights and high degree of semi‐crystallinity^[^ ^5^, ^6^ ^]^, although advances in multi‐material design^[^ ^7^ ^]^ have mitigated some of these limitations to drive recent developments in the bio‐electronic sector.^[^ ^8^ ^]^ Other approaches have instead blended insulating, thermosetting polymers (i.e., permanent covalent networks) with conductive fillers such as graphene, carbon nanotubes, or rigid metallic particles creating electrically conductive composites.^[^ ^9^, ^10^ ^]^ Although percolation thresholds < 1 wt.% can be reached for graphene and carbon nanotubes in some composites, obtaining optimized (plateau) conductivity values often requires significantly greater filler content.^[^ ^11^ ^]^ While this approach produces materials with good mechanical strength and modulus in the GPa‐range, they are not recyclable due to their permanent network structure.^[^ ^12^, ^13^ ^]^

Attention has turned toward replacing the permanent covalent bonds in conventional thermosets with dynamic covalent bonds to yield a dynamic covalent polymer network, or vitrimer.^[^ ^14^ ^]^ The resulting material can flow after application of a stimulus (typically heat) to activate bond exchange reactions and enable reprocessing or reshaping.^[^ ^15^ ^]^ This unique property makes vitrimers mechanically strong and chemically resistant like thermosets, but reconfigurable and recyclable like thermoplastics.^[^ ^16^ ^]^ In this space, there has been considerable interest to replace traditional epoxy composites due to their high volume usage in many industries, including electronics, with epoxy vitrimer composites.^[^ ^17^ ^]^ The most common synthetic approach for epoxy vitrimers is to install dynamic ester linkages within the epoxy network using anhydrides or carboxylic acids as hardening agents, which requires harsh reaction conditions for curing and the addition of an exogenous catalyst (i.e., additionally added) to render the subsequent material dynamic.^[^ ^18^, ^19^ ^]^ Together, these factors necessitate intricate manufacturing approaches, especially when including functional filler components.^[^ ^20^ ^]^

Solid conductive fillers have been successfully incorporated into vitrimer matrices,^[^ ^21^, ^22^, ^23^ ^]^ but the bulk electrical conductivity is typically well below that of the respective pristine filler component. On the other hand, using low‐melting‐point metal alloys, commonly referred to as liquid metals (LM), offers the prospect of creating high‐performance composites for reconfigurable electronics due to their high electrical and thermal conductivity, regenerative characteristics, and resistance to mechanical fatigue.^[^ ^24^, ^25^, ^26^ ^]^ However, using LM as an inclusion in high‐*T*_*g*_ vitrimer composites remains rare. The few examples of LMs in vitrimer matrices have displayed functional properties like thermal conductivity^[^ ^27^, ^28^ ^]^ but electrical conductivity, especially in mild processing conditions, was not achieved. LMs have primarily been added to low‐*T*_*g*_, elastomeric polymers or gels to afford soft, healable electronic materials with electrical conductivities several orders of magnitude greater than composites with carbon‐based fillers.^[^ ^29^, ^30^, ^31^, ^32^ ^]^ These types of soft LM composites have also been shown to be recyclable, when water soluble or thermoplastic elastomer matrices are used which leverage weaker physical crosslinks, in flexible or stretchable systems.^[^ ^33^, ^34^, ^35^ ^]^ Similarly, a polyimine vitrimer substrate with screen‐printed LM wiring has been demonstrated for soft and deformable devices; however, this material has a modest *T*_*g*_ near ambient temperature, low modulus, and a bi‐layer structure rather than a composite architecture with LM inclusions embedded in the matrix.^[^ ^36^ ^]^ Therefore, recyclable electrically conductive LM composites with plastic‐like qualities, including high *T*_*g*_ with high modulus and flexibility, remain underdeveloped and presents an opportunity for robust and recyclable materials for the reduction of E‐waste.

**Figure 1:**
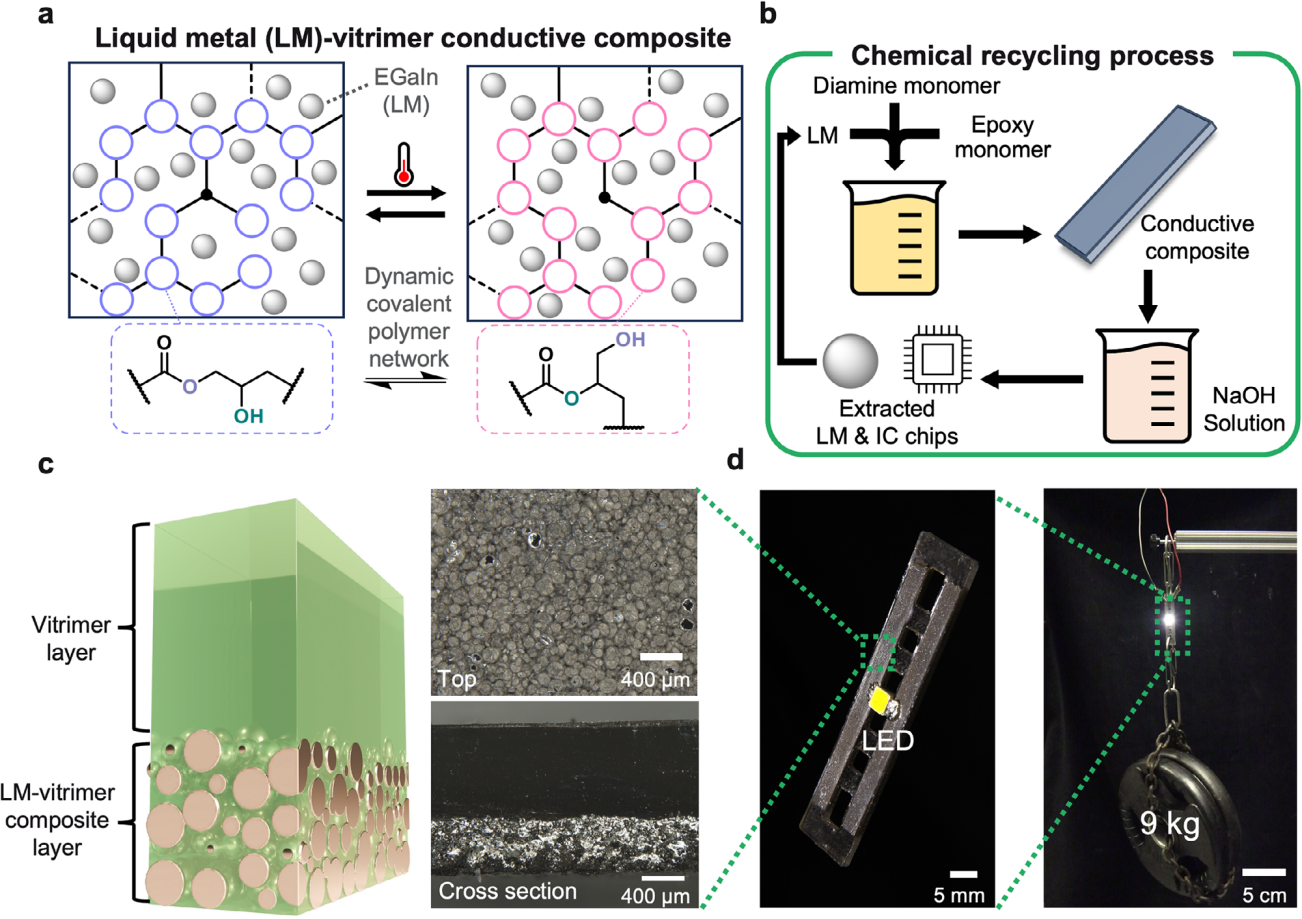
Liquid metal (LM)‐vitrimer electrically conductive composite and recycling process. a) Diagram of the dynamic covalent polymer network mechanism in the LM composite. b) Diagram of fabrication and chemical recycling of LM‐vitrimer conductive composite. c) Schematic of heterogeneous LM‐vitrimer composite structure (left) with top‐down and cross‐section microscopic images (right). d) Demonstration of 1.5 mm thick LM‐vitrimer composite sample supporting 9 kg of weight while an LED is on.

Here, we introduce recyclable and healable electronic materials through a LM‐vitrimer microdroplet composite which displays mechanical qualities of rigid thermosets yet recyclability through a dynamic covalent polymer network (**Figure** **1**a). These electrically conductive, plastic‐like composites show excellent thermomechanical properties using a mild curing process at 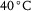 via ring‐opening polymerization of an ester‐based epoxy resin and amine hardener with LM droplets added in situ during polymerization (Figure 1b). During the curing procedure, the eutectic Ga‐In (EGaIn) based LM droplets settle so that each side has either a conductive or insulating functionality (Figure 1c). The resulting material exhibits high *T*_*g*_ (≈130°C) and high elastic modulus (≈1 GPa), good solvent resistance, high electrical conductivity (2.0 x 10^5^ S m^−1^), reconfigurability or shape memory, and recyclability. Further, these characteristics are robust even under damage, with material healing and electrical self‐healing enabled by the dynamic nature of the vitrimer and LM inclusions. Unlike previous LM incorporated composites which have focused on permanent covalent networks or physically cross‐linked networks for soft devices, our LM‐vitrimer composites enable a unique combination of electrical conductivity, robust thermomechanical performance, high modulus, and recyclability without loss of electrical conductivity under high loads or deformation (Figure 1d). This LM‐vitrimer composite establishes a pathway towards fully recyclable, mechanically robust, and reconfigurable and healable electronics, thus advancing the field of electronic materials.

## 2 Results and Discussion

### 2.1 Vitrimer Synthesis and Electrical/Mechanical Characterization

**Figure 2:**
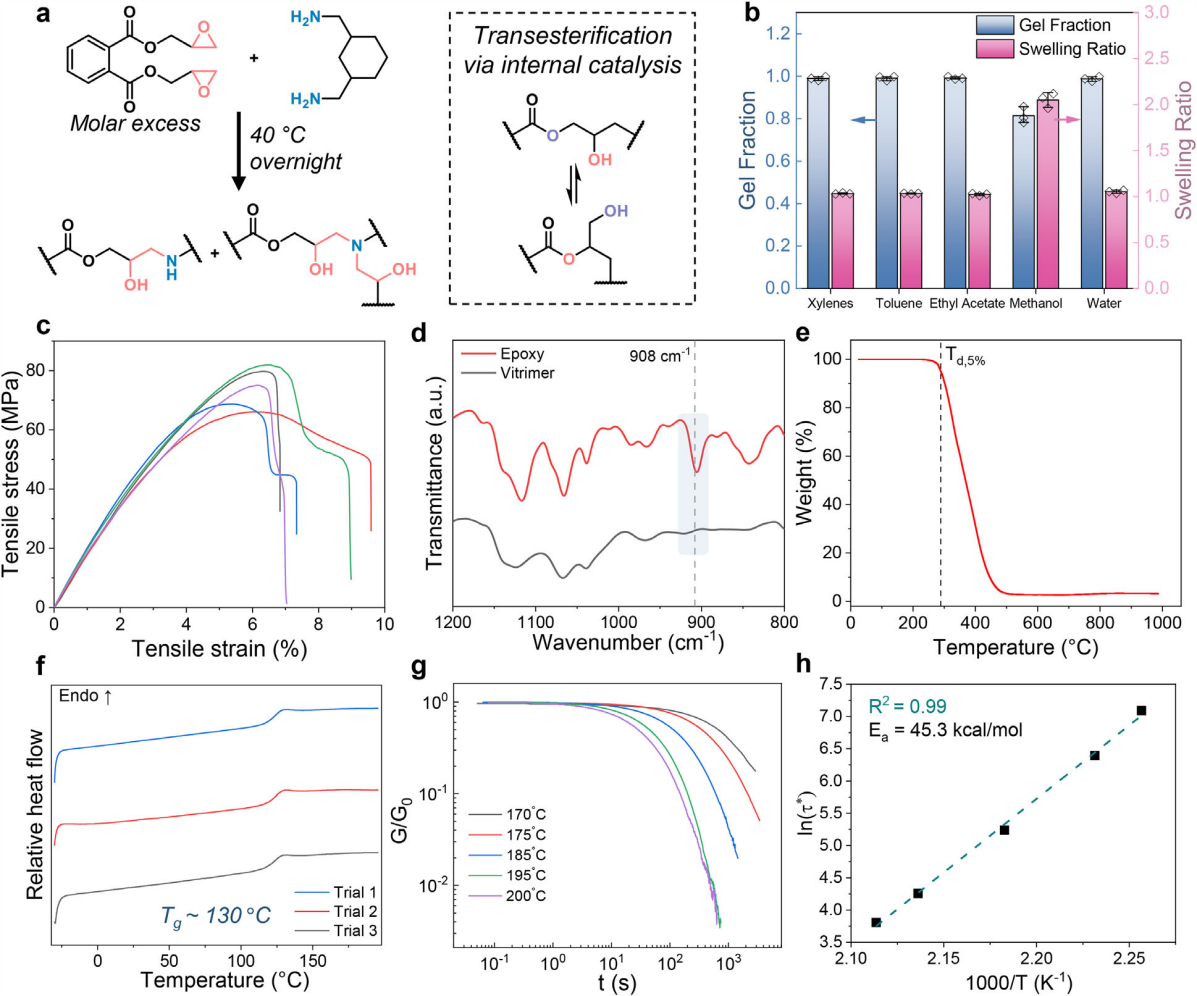
Pristine vitrimer property characterization. a) Left: Ring opening polymerization of ester‐based epoxy and amine. Right: Dynamic transesterification reaction activated by internal amine catalyst. b) Gel fraction and swelling ratio of the vitrimer in five different solvent tests. *n* = 3 from individual samples are presented as scatter symbols and data bars represent mean ± s.d. c) Stress–strain curves of five individual vitrimer samples. d) Fourier‐transform infrared spectroscopy (FTIR) of the epoxy and vitrimer. e) Thermogravimetric analysis (TGA) of the vitrimer. f) Differential scanning calorimetry (DSC) for three individual vitrimer samples. g) Stress relaxation characterization of the vitrimer on a log–log plot for different temperatures, where *G* and *G*_0_ denote the modulus at time and original modulus of the vitrimer. h) Semi‐logarithmic plot fitting an Arrhenius equation to τ* versus inverse temperature with *R* ^2^ and activation energy for transesterification (*E*_*a*_).

It is important to understand the thermomechanical and rheological behavior of the vitrimer matrix before incorporating LM for subsequent composite fabrication. Pure ester‐based epoxy vitrimer was synthesized by reacting diglycidyl phthalate (DP) with 1,3‐bis(aminomethyl)cyclohexane (AH) via ring‐opening polymerization of epoxy (**Figure** **2**a left). Although the epoxide is in molar excess relative to the primary amine (‐NH_2_), each primary amine unit (‐NH_2_) in AH can theoretically react twice with an epoxide moiety of DP. However, the steric hindrance around a secondary amine is much higher than that around a primary amine, reducing its ability to attack a second epoxide. Nevertheless, the secondary amines that do react with epoxide units serve as dynamic covalent cross‐links to form a transient polymer network. The secondary (after one addition) and/or tertiary (after two additions) amines in the formed network also serve as built‐in internal catalysts^[^ ^37^ ^]^ for subsequent transesterification reactions during remolding (Figure 2a right). In contrast to epoxy‐acid vitrimers which rely on carboxylic acids as hardening agents,^[^ ^38^, ^39^ ^]^ the built‐in internal ester design coupled with an amine hardener avoids high‐temperature curing (e.g., 180 °C) that is typical to epoxy vitrimer resins. It also advantageously avoids the use of an exogenous catalyst.^[^ ^40^ ^]^ This enables us to cure and process our composites at low temperatures (40 °C), yet still achieve a high *T*_*g*_, elastic modulus, and electrical conductivity.

To assess the relative degree of cross‐linking, gel content and swelling ratio were experimentally determined and presented in Figure 2b (experimental details can be found in the Experimental Section). Briefly, a 10 mm pre‐weighed disk sample was swollen in the corresponding solvent for 7 days. The sample was then removed, and the residual solvent on the surface was wiped with a Kimwipe prior to weighing. Besides methanol that may be able to swell and partially dissolve the polymer well, we observed a high gel fraction in most solvents which indicates the as‐synthesized polymer is sufficiently cross‐linked. We tested the swelling behavior under elevated temperature/reflux conditions (140 °C, 24 h) with xylenes in a Soxhlet extractor and observed negligible difference compared to the non‐heated test, confirming stability and crosslink robustness of the vitrimer network (Figure S1, Supporting Information). This was further confirmed with mechanical testing under tension, where the ultimate tensile strength ranged between 60 and 80 MPa, further supporting the high crosslinking density (Figure 2c).

With the support of Fourier‐transform infrared (FTIR) spectroscopy (Figure 2d), the absence of a peak at 908 cm^−1^ indicated that unreacted epoxide was undetectable (FTIR spectrum of full measurement range is presented in Figure S2, Supporting Information). Thermogravimetric analysis (TGA) (Figure 2e) revealed that the as‐synthesized vitrimer has a *T*_*d*, 5%_ at ${298\;}^{\circ }C$ which is above the required working temperature in most applications. Differential scanning calorimetry (DSC) of the sample also showed a *T*_*g*_ around 130 °C suggesting a highly cross‐linked network (Figure 2f). To assess the dynamic ester bonds, we further characterized the topology freezing temperature (*T*_*v*_ ≈ 165 °C) of the vitrimer by measuring the strain response under constant stress (200 kPa) as a function of temperature through dynamic mechanical analysis (DMA) in the single cantilever mode at a heating rate of 2 °C min^−1^ (Figure S3, Supporting Information).^[^ ^41^ ^]^

Stress relaxation experiments were performed on a rheometer at different temperatures to analyze the bulk flow behavior from dynamic bond exchange by applying 1% deformation to the material and monitoring the modulus over time. A conventional polymer network or thermoset, such as epoxy, that is used in electronic materials usually cannot relax the stress under any conditions due to permanent covalent bonding.^[^ ^42^ ^]^ With a dynamic covalent bond incorporated into the network, the stress can be relaxed over time when the bond is activated under certain triggers, such as elevated temperature. Transesterification reactions typically need to be activated at high temperatures^[^ ^19^ ^]^ so the material was assessed between 170 °C and 200 °C to determine an optimal temperature with a reasonable relaxation time for subsequent remolding of the vitrimer system. The stress relaxation data is plotted on log–log scales in Figure 2g (raw data is presented in Figure S4, Supporting Information), and is fit with a stretched exponential or Kohlrausch–Williams–Watts (KWW) function to determine characteristic relaxation time (τ*),^[^ ^43^ ^]^ which are presented in Figure S5 (Supporting Information). Arrhenius equation was used to understand the temperature dependence of the transesterification reaction rate and determine the activation energy of the transesterification reaction in the bulk sample by plotting ln(τ*) against 1000/*T* (Figure 2h). The calculated activation energy (*E*_*a*_) is 45.3 kcal mol^−1^, which is comparable to previous literature values for epoxy vitrimers.^[^ ^44^, ^45^ ^]^ With results from stress relaxation experiments, we conducted remolding tests at 170 °C under 1.5 metric tons for 30 min up to four cycles. The remolded samples show good mechanical integrity (Figure S6, Supporting Information). Due to the dynamic nature of ester bonds within the vitrimer, the material can undergo healing, remolding, and chemical recycling (as seen below).

### 2.2 LM‐Vitrimer Conductive Composite Mechanical and Electrical Properties

We utilized EGaIn LM inclusions to achieve high electrical conductivity and flexibility in the vitrimer‐based composite materials. The alloy EGaIn (75 wt.% gallium, 25 wt.% indium) was selected for its low toxicity and high electrical conductivity while retaining a sub‐ambient melting temperature.^[^ ^32^ ^]^ Unlike fixed conductive paths in solid fillers, liquid conductive networks can be reconfigured when deformed, offering stable electrical responses.^[^ ^46^ ^]^

Incorporation of LM into the vitrimer polymer matrix was accomplished by a shear mixing procedure. First, the designated volume fraction (*ϕ*_*LM*_) of LM was mixed with the viscous epoxy resin using a planetary mixer. During the mixing procedure, the shear stress inside the epoxy monomer breaks the bulk LM into microdroplets (≈80μm). The diamine hardener was incorporated into reaction mixture and then this mixture was poured into a polydimethyl siloxane (PDMS) mold followed by mild curing in a convection oven (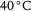, 3 h).

**Figure 3:**
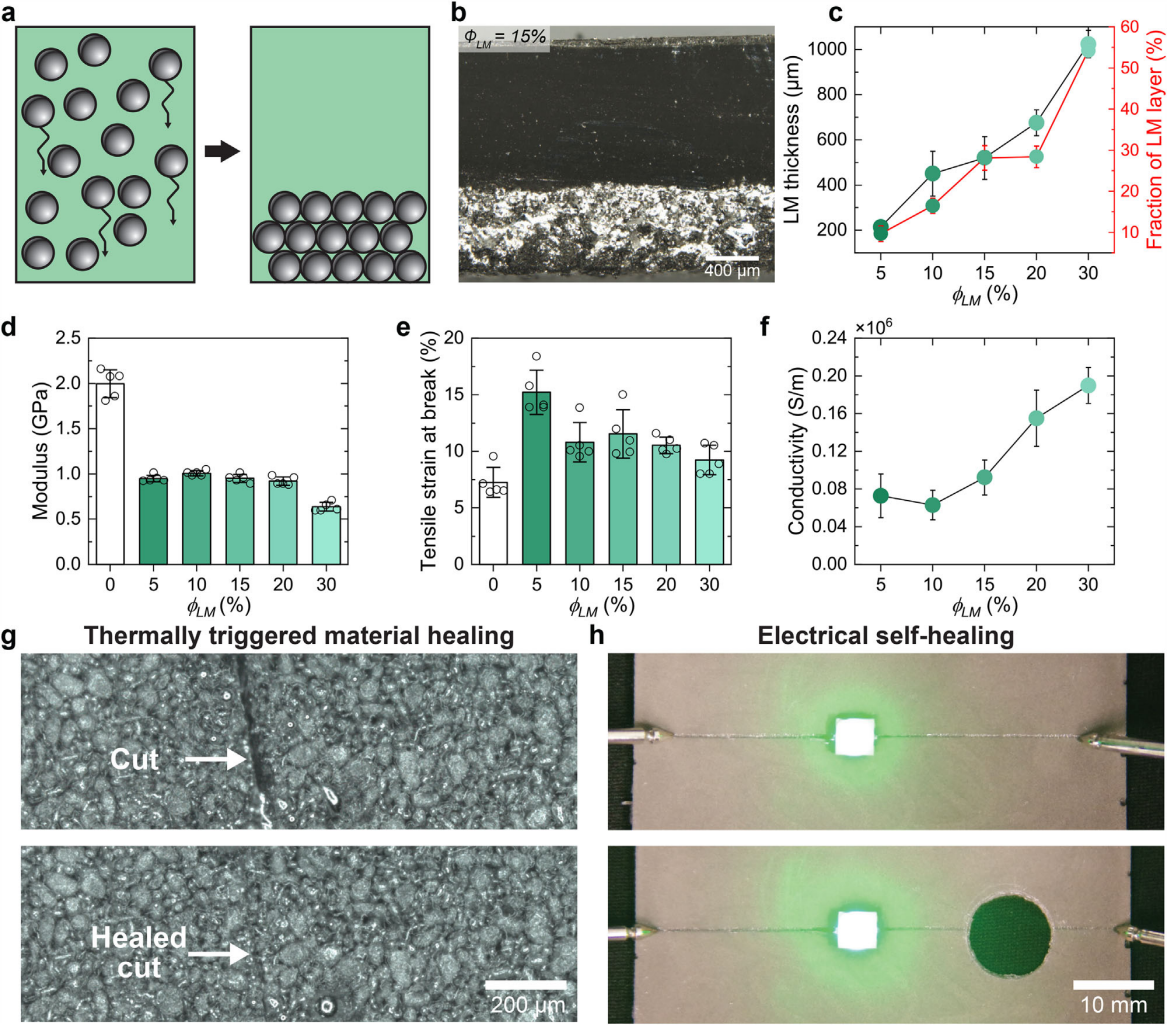
LM‐vitrimer conductive composite characterization and healing. a) Schematic showing LM microparticle sedimentation in uncured vitrimer. b) Cross‐sectional micrograph of a *ϕ*_*LM*_ = 15 vol.% LM‐vitrimer composite. c) Thickness of the LM layer in LM‐vitrimer composites as a function of *ϕ*_*LM*_. *n* ≥ 5 measurements for each condition with data presented as mean ± s.d. d) Modulus and e) Tensile strain at break for LM‐vitrimer composites as a function of *ϕ*_*LM*_. *n* = 5 from individual samples are presented as scatter symbols and data bars represent mean ± s.d. f) Electrical conductivity of LM‐vitrimer composites as a function of *ϕ*_*LM*_. *n* = 4 from individual samples and data are presented as mean ± s.d. g) Thermally triggered material healing of a surface cut on an *ϕ*_*LM*_ = 30% LM‐vitrimer composite (full images are presented in Figure S11, Supporting Information). h) Electrical self‐healing of an *ϕ*_*LM*_ = 30% LM‐vitrimer composite where an LED continues to function while a hole is punched through the conductive trace powering the LED (images and schematics are presented in Figure S12, Supporting Information).

During the curing procedure, sedimentation of the LM microdroplets occurs due to the greater density of LM, as shown in **Figure** **3**a,b. Sedimentation of the LM results in a heterogeneous structure in the composite such that one side is electrically conductive (LM phase) and the other is insulating (vitrimer phase). Additional optical microscopy images for composites with different liquid metal volume ratios are shown in Figure S7 (Supporting Information). The localized concentration of LM microdroplets within the specified volume is advantageous because it substantially reduces the distance between droplets, consequently effectively lowering the percolation threshold. Theoretically, inclusion volume fractions (*ϕ*) in excess of 25% are required for a 50% probability in forming a percolated network.^[^ ^47^, ^48^ ^]^ However, due to the localized nature of the droplets, electrical conductivity was achieved with a minimal LM volume fraction (*ϕ*_*LM*_) as low as 5%. Because of the intrinsic formation of a non‐LM‐containing layer at the top, an electrically insulating layer naturally forms during the fabrication. By increasing *ϕ*_*LM*_, the thickness of the sedimented LM layer increases. As *ϕ*_*LM*_ increases from 5 to 30%, the LM layer accounts for a greater fraction of the total film thickness, increasing gradually from 10 to 55% (Figure 3c). Further, multilayer structures can be created using sedimentation of LM droplets coupled with subsequent film casting. This includes A‐B A‐B A‐B (A: LM‐vitrimer composite, B: Vitrimer) with different *ϕ*_*LM*_ in each A sub‐component (Figure S8, Supporting Information) as well as A‐B B‐A architectures with the same *ϕ*_*LM*_ in each A sub‐component (Figure S9, Supporting Information). This enables the fabrication of film architectures that resemble printed circuit board structures, with electrically conductive and insulating layers inherently formed during the manufacturing process.

Liquid metal inclusions inside the solid polymer matrix impact the modulus and ductility of the composite materials. The mechanical properties of each LM‐vitrimer composite were evaluated under tension using a universal testing machine (all stress–strain curves are presented in Figure S10, Supporting Information). The pristine vitrimer shows a modulus of 1.8 GPa, while the addition of LM inclusions decreases the modulus at all *ϕ*_*LM*_ (Figure 3d). The modulus decreases once LM is introduced at *ϕ*_*LM*_ = 5% and then remains relatively constant, within the range of the error bars, around 1 GPa until *ϕ*_*LM*_ = 20%. The modulus then decreases for *ϕ*_*LM*_ = 30%, where the modulus reaches the minimum value of 0.6 GPa. This decrease can be attributed to the liquid nature of the LM inclusions and the sedimentation process. As *ϕ*_*LM*_ increases, the total thickness fraction of the sedimented LM layer increases at a rate faster than the volume loading. Specifically, at *ϕ*_*LM*_ = 30%, the LM/vitrimer layer occupies approximately 55% of the total composite thickness, whereas at *ϕ*_*LM*_ = 20%, the LM/vitrimer layer accounts for 28% (Figure 3c). This suggests that the sedimentation layer is not completely dense, but instead forms a foam‐like structure within the rigid vitrimer that would be expected to soften the composite.^[^ ^49^ ^]^ Adding LM droplets increases the tensile strain at break of the LM‐vitrimer composites relative to the pristine vitrimer (Figure 3e). At *ϕ*_*LM*_ = 5%, the strain at break is approximately twice that of the pristine vitrimer. For 10% ≤< *ϕ*_*LM*_ ≤< 20%, the strain at break is lower than *ϕ*_*LM*_ = 5%, but remains relatively constant, decreasing slightly for *ϕ*_*LM*_ = 30%. Nonetheless, all composite samples exhibit greater extensibility than the pristine vitrimer.

The electrical properties of the LM‐vitrimer composite were also investigated. The LM‐vitrimer composite was first activated using an embossing method similar to previous studies.^[^ ^31^, ^34^, ^50^ ^]^ Since the vitrimer matrix is rigid at room temperature, it was first softened with gentle heating before embossing at a designated location. A custom‐made four‐point probe measurement was then used to measure the conductivity of the composite. The composites became electrically conductive at small loadings of LM, where the *ϕ*_*LM*_ = 5% composite displayed an electrical conductivity of 0.7 × 10^5^ *S m* ^−1^ (Figure 3f). This is notable, considering LM microdroplet‐based composites with uniform inclusion distributions typically require 20–50% *ϕ*_*LM*_ to become conductive.^[^ ^34^, ^50^, ^51^, ^52^ ^]^ A further increase of *ϕ*_*LM*_ increases the conductivity. At 30% *ϕ*_*LM*_, the electrical conductivity increases to 2 × 10^5^ *S m* ^−1^.

The composite also exhibits healing capabilities due to the dynamic ester bonds and reconfigurable LM droplet network. To demonstrate material healing, a razor blade was used to cut the surface of a composite with *ϕ*_*LM*_ = 30% (Figure 3g; Figure S11, Supporting Information). The damage was then repaired through a thermal trigger, enabled by the Joule heating capabilities of the composite. As power was applied the composite heated and reconfigured to heal the cut in 10 min, as shown in the bottom micrograph of Figure 3g. Beyond material healing, the LM network allows the composite to autonomously restore its electrical conductivity. This was demonstrated by fabricating an LED circuit, where selective embossing activated LM droplets to form a conductive trace in a *ϕ*_*LM*_ = 30% composite (Figure 3h, top, and Figure S12a, Supporting Information). When severe damage was introduced by punching a hole through the composite, the LM network instantly reconfigured around the damage, maintaining electrical conductivity and ensuring continuous LED illumination (Figure 3h, bottom, and Figure S12b, Supporting Information).

Together, these results demonstrate that the LM‐vitrimer composite functions as a rigid plastic with high electrical conductivity. Further, these characteristics are robust even under damage, with material healing and electrical self‐healing enabled by the dynamic nature of the vitrimer and LM inclusions.

### 2.3 LM‐Vitrimer Composite Strength, Shape Memory, and Recycling

**Figure 4:**
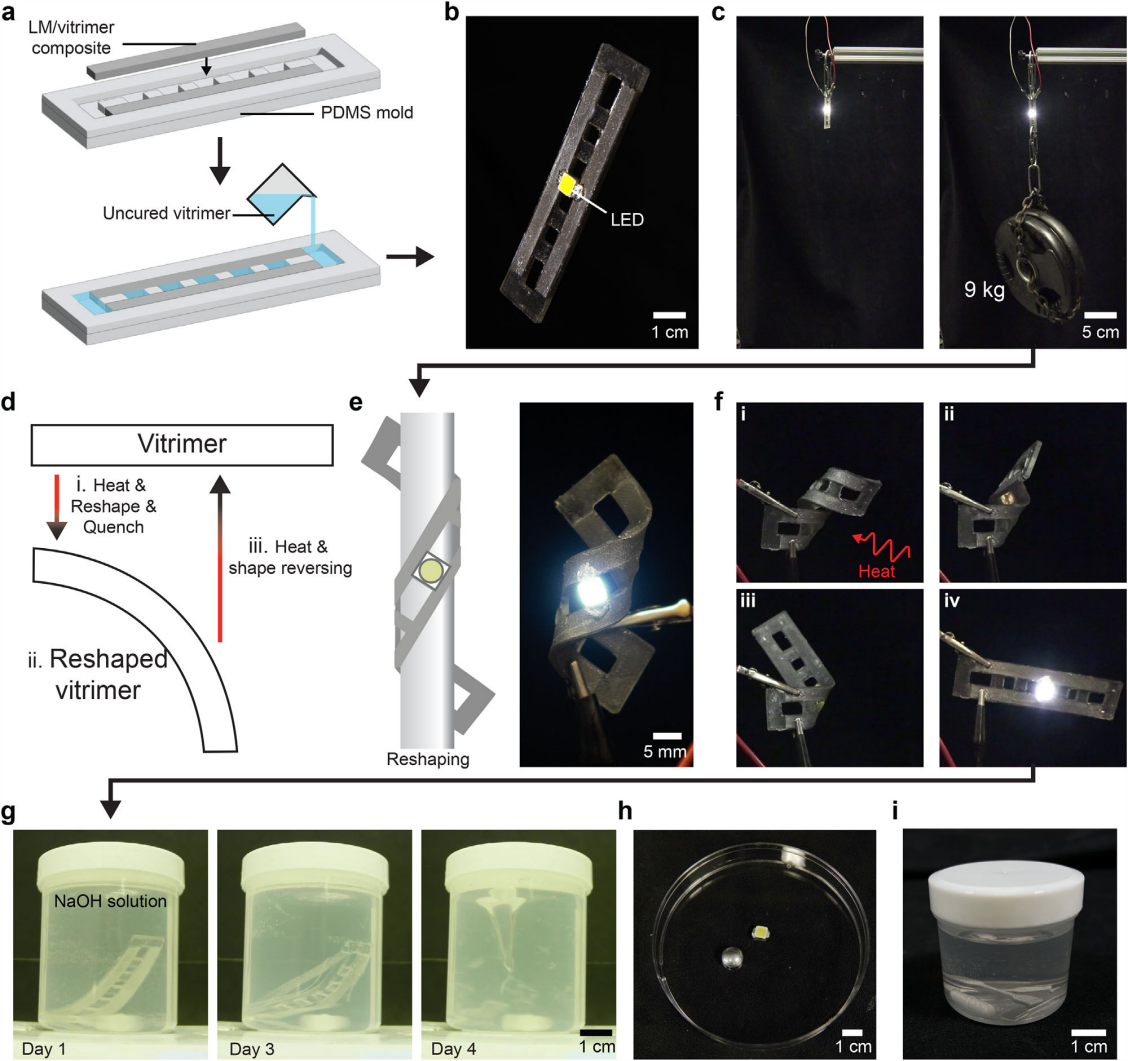
Demonstration of LM‐vitrimer conductive composite. a) Fabrication of LM‐vitrimer composite LED device. b) Photograph of the fabricated LM‐vitrimer composite LED device. c) LM‐vitrimer conductive composite holding a 9 kg weight while powering an LED. d) Schematic of the shape memory behavior. e) Schematic (left) and image (right) showing reshaping of the LED device into a cylindrical shape. f) Image sequence of the shape memory recovery by applying heat. g) Image sequence of LM‐vitrimer composite disintegrating in 5M NaOH solution. h) Extracted LM and LED chip from the degraded composite. i) Control epoxy (without ester functionalities) LM composite showing no degradation after immersion in 5M NaOH solution for 3 days.

The LM‐vitrimer conductive composite possesses qualities that are intrinsic to both thermoplastic and thermoset materials. One of the key advantages is its superior mechanical strength and rigidity owing to its highly cross‐linked network. To demonstrate this, we fabricated a simple circuit with a single LED. To make a distinct anode and cathode for the LED, we first prepared a bar‐shaped LM‐vitrimer conductive composite (*ϕ*_*LM*_ = 20%) as shown in **Figure** **4**a. Next, we placed two LM‐vitrimer conductive composites inside a PDMS mold and poured uncured vitrimer to join them. The sample was then cured and subsequently extracted from the mold (Figure 4b). The fully cured multi‐material composite displayed excellent structural stability (no delamination), which we hypothesize is facilitated by dynamic transesterification reactions at the interface of the already cured LM‐vitrimer composite and the newly poured vitrimer, i.e., effectively leading to healing behavior. This adhesive‐like healing behavior is distinct from that of common epoxy materials, which require a surface preparation procedure of the already cured epoxy surface before adding additional resin. This feature of the epoxy vitrimer enables the bulk device to have spatial control over electrical conductivity while maintaining uniform mechanical strength throughout the material.

The fabricated device was then used as a multifunctional, load‐bearing electrical conductor as more than 9kg of weight plates were held while the LED in contact with the LM‐vitrimer was turned on (Figure 4c; Movie S1, Supporting Information). The LM‐vitrimer conductive composite sample was able to withstand the 9kg weight successfully despite a thickness of only 1.5 mm. This impressive strength further highlights the seamless interfacial bonding between the conductive composite and the newly poured vitrimer layer. Due to fast curing kinetics under mild conditions, the LM‐vitrimer composites show the potential to be used as on‐demand conductive materials that could be deployed in various field applications.

The LM‐vitrimer composite also shows thermally triggered shape‐memory behavior (Figure 4d). This is due to the combination of its cross‐linked structure paired with flexible (aliphatic linkers) and stiff (aromatic/cyclohexane moieties) segments.^[^ ^53^, ^54^ ^]^ To demonstrate the shape‐memory effect, the same sample used for the weight‐hanging demonstration was wrapped around a cylindrical rod while being heated above the glass transition temperature (Figure 4e left). Then, the composite was cooled to room temperature to fix the coiled shape (Figure 4e, right). To recover its original shape, the sample was heated above its *T*_*g*_ using a heat gun while the LED was on (Figure 4f and Movie S2, Supporting Information). The original shape was recovered while the LED remained on, indicating that the electrical conductivity was preserved during the shape‐memory cycle.

Additionally, Joule heating through the conductive LM network enables reshaping and reprocessing of the composite. This was demonstrated using a *ϕ*_*LM*_ = 30% LM‐vitrimer composite, where applying a voltage of 5 V and a current of 5 A through a power supply generated heat inherently, allowing the flat composite to be reprocessed (Figure S13a, Supporting Information). After heating for 30 s, the composite softened and was shaped into an arc. Then, the power supply was turned off, the composite cooled below its *T*_*g*_, and regained rigidity. Next, the power supply was switched on to trigger shape recovery of the composite back to the flat state. The whole process was recorded through an Infrared (IR) camera to observe heat generation and dissipation as the composite was reshaped (Figure S13b, Supporting Information).

We subsequently investigated the degradability of the composite by chemical recycling. When the LM‐vitrimer composite reaches end‐of‐life, valuable resources inside the composite must be retrieved. Because of the presence of ester bonds in the matrix, the composite could be degraded by base‐catalyzed hydrolysis using an aqueous sodium hydroxide solution (5 M). Here, the NaOH solution simultaneously serves another purpose. The strongly alkaline solution also removes the gallium oxide shell on the LM microdroplets, which increases the LM fluidity and aids in reclamation of the LM. To demonstrate bulk degradation of the device and recovery of components, the same sample that was used for both the weight hanging and shape memory demonstration was submerged in a 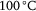 5 M NaOH solution with a magnetic stir bar stirring at 1000 RPM (Movie S3, Supporting Information). The LM‐vitrimer composite mostly retained dimensional stability until day 2, but on day 3 the sample fractured into several pieces with noticeable release of LM droplets (Figure 4g; Movie S3, Supporting Information). On day 4, the vitrimer matrix was fully disintegrated, and both LM and LED components were easily extracted from the solution using a 200 mesh sieve as shown in Figure 4h. As a control, an epoxy‐based composite without ester bonds (i.e., conventional thermoset) was submerged in an identical alkaline solution. However, the control sample did not display any sign of deterioration and retained its structural integrity (Figure 4i), indicating that the dynamic ester bond is essential to chemically recycle the composite material.

### 2.4 LM‐Vitrimer Reconfigurable Circuit Board

**Figure 5:**
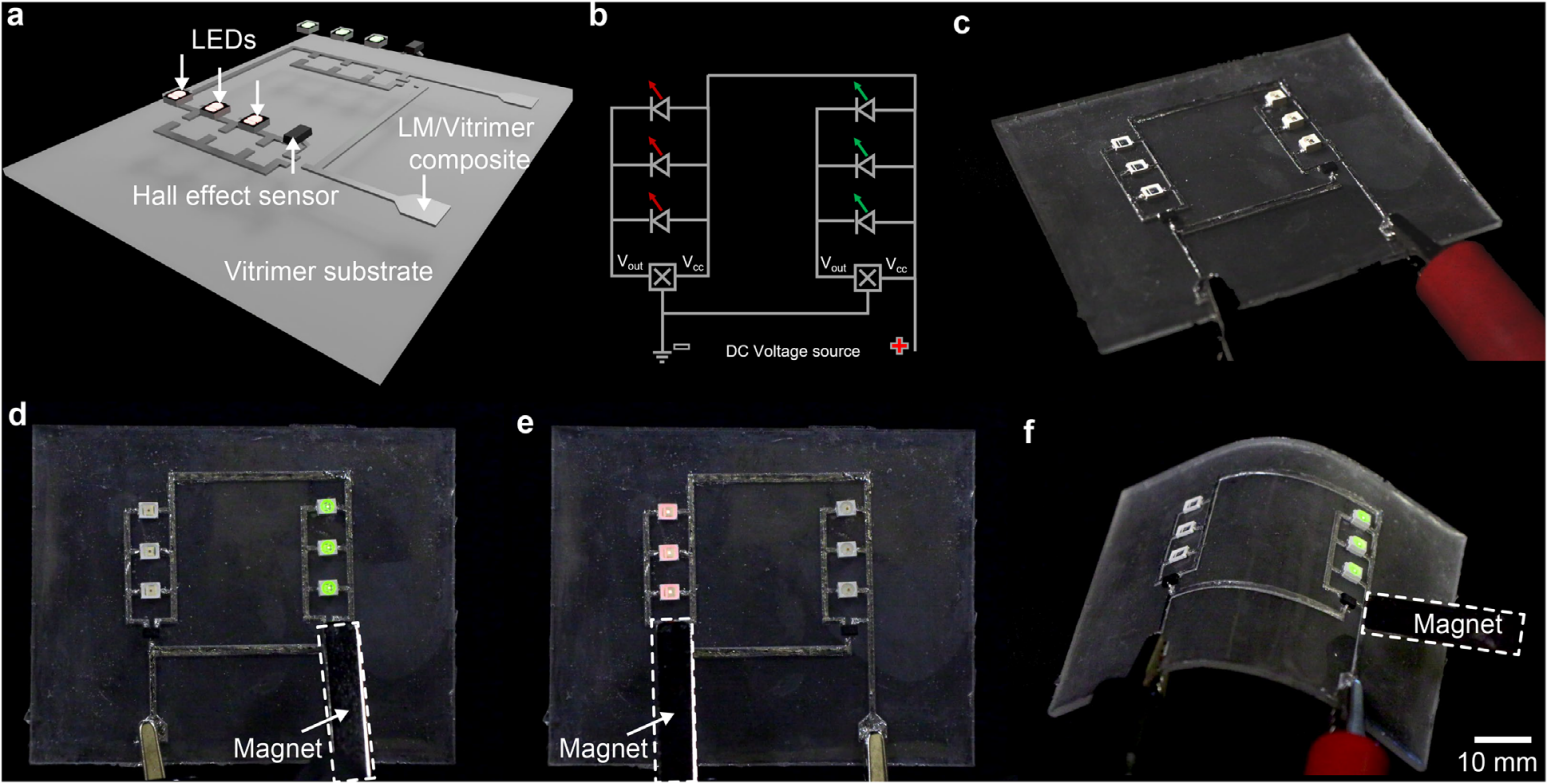
Demonstration of LM‐vitrimer circuit. a) Schematic of Hall effect sensor circuit with LEDs connected by LM‐vitrimer composite interconnects assembled on pure vitrimer. b) Circuit design with two Hall effect sensors and six LEDs (three each of red and green). c) Fabricated circuit with electrically conductive interconnects, created by abrasion force on the LM‐vitrimer composite surface. d) Photograph of the circuit when the green LEDs lit up due to the magnetic field applied on the connected Hall effect sensor. e) Photograph with red LEDs lit up. f) The functioning of the circuit demonstrated after being reconfigured by heat application. Images in (c) to (f) follow scale bar in (f).

To demonstrate potential applications of our material for next generation sustainable electronic devices, we created a fully vitrimer based circuit board (**Figure** **5**a). The circuit board is a transparent vitrimer sheet with dynamic ester bonds. The circuit layer is a screen printed LM‐vitrimer composite that integrates two sets of LEDs and Hall effect sensors in parallel (a circuit schematic is shown in Figure 5b while a top‐down view of the fabricated circuit board is shown in Figure 5c).

The Hall effect sensor outputs a voltage (*V*_*out*_) that changes with magnetic field strength. When there is no magnet, the potential difference between *V*_*out*_ and *V*_*cc*_ is insufficient to turn on the LEDs (Figure 5c). However, as the magnetic field increases, the potential difference between *V*_*out*_ and *V*_*cc*_ increases, eventually powering the LEDs as shown in Figure 5d and Movie S4 (Supporting Information). As each set of LEDs is connected in a parallel circuit configuration, both the left and right sides can be operated independently. When the magnet moves from the right side Hall effect sensor to the left side sensor, LEDs at the right side dim and eventually shut off and the left side LEDs begin illuminating (Figure 5e).

The reconfigurability of the circuit and its ability to maintain electrical functionality after being reshaped is also demonstrated. Here, heat was applied to reshape the circuit, and the circuit continued to function after being reshaped (Figure 5f). These demonstrations suggest that our vitrimer and LM‐vitrimer composite can effectively create electronics that integrate rigid circuit components, offering potential for future sustainable electronic devices.

## 3 Conclusion

This dynamic LM‐composite provides a pathway toward fully recyclable and mechanically robust electronics, thus advancing the field of electronic materials. Here, the LM‐vitrimer composite offers several advantages over conventional thermoset‐based conductive composite materials that incorporate solid fillers. These features include cohesive structural integrity and mechanical robustness combined with excellent electrical conductivity. Moreover, the high conductivity was not compromised under various operational conditions. A vitrimer‐based circuit board remained fully operational despite significant stress, deformation, and thermally triggered shape‐memory transformations, highlighting its potential as an advanced composite material. The electronic device was chemically recycled via mild hydrolysis leading to recovery of critical components from the matrix, including the LM and LED. Although the vitrimer matrix was effectively depolymerized, the byproducts were not re‐polymerizable. Thus, future work is aimed at recovering functional vitrimer components,^[^ ^55^ ^]^ which may be used to subsequently re‐synthesize pristine materials in a closed‐loop process. We anticipate that the structure‐property scope can also be greatly expanded by tuning the material composition using an array of commercially available resin components and hardeners. Unlike existing LM composites, our LM vitrimer material has unique combinations of high modulus, flexibility, and electrical durability while being recyclable. The use of a vitrimer network with LM droplets, rather than the permanent covalent networks typical of traditional thermosets or the physical networks found in thermoplastic elastomers, enables our unique combination of electrical conductivity, robust thermomechanical performance, and recyclability – which are key attributes for developing plastic‐like, recyclable circuit boards. This work substantially advances efforts toward creating a sustainable economy for electronic materials.

## 4 Experimental Section

### Vitrimer Sample Fabrication

Diglycidyl *o*‐phthalate (provided by Nagase & Co. LLC) and 1,3‐bis(aminomethyl)cyclohexane (*cis*‐ and *trans*‐ mixture, Fisher Scientific) were mixed in 1:0.625 ratio using a planetary mixer (FlackTek) at 2000 RPM (4 min, 50 Pa). The mixuture was then pour into a PDMS mold and cured in a convection oven (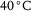) for 7 h to obtain a cross‐linked sample.

### LM‐Vitrimer Composite Fabrication

LM (Eutectic gallium indium alloy, Ga/In in the weight ratio of 3:1) was first added into the diglycidyl *o*‐phthalate (provided by Nagase & Co. LLC) with calculated *ϕ*_*LM*_ to the total volume of the final composite (0–30 vol.%). After hand mixing the LM and diglycidyl *o*‐phthalate mixture for 3 min, to make the LM microdroplets 2000 RPM, the mixture was placed in a planetary mixer (FlackTek) and mixed at 2000 RPM (1 min, 50 Pa). Then, 1,3‐bis(aminomethyl)cyclohexane (*cis*‐ and *trans*‐ mixture, Fisher Scientific) was added to the homogenized mixture. The weight of 1,3‐bis(aminomethyl)cyclohexane was calculated on the basis of the molecular ratio between diglycidyl *o*‐phthalate where the molar ratio between diglycidyl *o*‐phthalate and 1,3‐bis(aminomethyl)cyclohexane is 1:0.625. Finally, LM and diglycidyl *o*‐phthalate/1,3‐bis(aminomethyl)cyclohexane solution was poured into a PDMS mold and cured in the convection oven (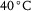) for 7 h to obtain a cross‐linked film.

### Thermomechanical and Chemical Characterization

Solvent swelling tests for gel content and swelling ratio were conducted by submerging samples in various solvents. Vitrimer samples were cut into 8 mm round disks with 1.5 mm thickness. The original mass of each sample was recorded as *m*_*original*_. Each sample was then submerged for 7 days in a six dram vial containing 8 mL solvent. The swelled sample was removed from the solvent and the surface was lightly dried with a Kimwipe and the corresponding mass was measured, denoted as *m*_*swell*_. Then each swelled sample was dried in vacuum oven at 100 °C under dynamic vacuum for 2 days until no more mass change was observed. The dried sample mass was recorded as *m*_*dried*_. The following equations are used to calculate gel content and swelling ratio:

(1) $$Gel\;content=\frac{m_{dried}}{m_{original}}$$

(2) $$Swelling\;ratio=\frac{m_{swell}-m_{dried}}{m_{dried}}\frac{\rho_{sample}}{\rho_{solvent}}+1$$

*m*_*original*_, *m*_*swell*_, and *m*_*dried*_ denote the weight of sample before swelling, after swelling and tap dried, and after fully dried, respectively. ρ_*sample*_ and ρ_*solvent*_ are the density of original sample and solvent, respectively.

For the heated swelling tests, xylenes was chosen as the solvent since it has a boiling point above the *T*_*g*_ of the vitrimer. Three pieces of samples were placed in a stapled filter paper in the Soxhlet extractor. 150 mL solvent was brought to boil by a 200 °C sand bath. A Findenser was attached to the extraction system to constantly condense the solvent back to the system. The Soxhlet extraction was then run for 24 h. After completing the heated swelling test, drying and measurement procedures were the same to those used for the non‐heated samples.

Thermal gravimetric analysis (TGA) thermograms were performed using a TGA 550 Thermogravimetric analyzer (TA instruments). Thermograms were recorded under a nitrogen atmosphere at a heating rate of 20 °C min^−1^ from 25 to 1000 °C. Decomposition temperatures were reported at the 5% weight‐loss‐temperature (*T*_*d*_, _5__%_).

Differential scanning calorimetry (DSC) thermograms were collected on TA Discovery DSC 2500. Thermograms were obtained in 40 μL aluminum pans from −30 to 200 °C at a heating rate of 20 °C min^−1^ for two heating/cooling cycles unless otherwise specified. The (*T*_*g*_) was determined by the region of greatest slope change in the second heating cycle of DSC.

Infrared spectra were obtained with a ThermoFisher Scientific Nicolet iS5 fourier transform infrared (FTIR) spectrometer with an iD7 ATR (Attenuated Total Reflection) accessory equipped with a diamond cell in attenuated total reflection mode. Samples were scanned at operating wavelengths in the range between 4000 and 600 cm^−1^ with 0.5 cm^−1^ wavenumber resolution, and each measurement consisted of 32 scans at room temperature.

Rheological experiments were performed on HR 20 Discovery Hybrid Rheometer equipped with 8 mm parallel plates and an environmental test chamber to ensure stable temperature during measurement. Before any measurements were performed, the system was initialized and calibrated properly. The gap size was typically set at around 1.00 mm, similar to the thickness of the polymer discs being measured. When necessary, a small normal force of 1 N was applied to reduce slippage between the plates and the sample.

Stress relaxation experiments were performed by applying a 1% strain on the sample, and measuring the modulus (*G*(*t*)) over time. The experiments were conducted at temperatures between 170 to 200 °C. The stress relaxation experiments were performed starting from the lowest temperature (170 °C) and incrementally increasing the temperature for each successive measurement. Before each new run, the sample was left for 5 min at the new temperature to ensure thermal equilibrium. The characteristic relaxation time (τ*) was determined by a stretched exponential or Kohlrausch–Williams–Watts (KWW) function. Arrhenius plots were then constructed by plotting ln(τ*) as a function of the reciprocal temperature (1000/*T*) to determine the activation energy (*E*_*a*_).

### Microscopy

The optical micrographs of the composite microstructure in top‐view and cross‐sectional configurations were obtained using a Zeiss Axiozoom V.16 microscope.

### Mechanical Characterization

Samples were fabricated with a dogbone die according to ASTM D638 type V standard, size uniformly scaled to 50%. Mechanical properties vitrimer and LM‐vitrimer composites were tested on an Instron 5944 mechanical testing machine in tension mode using an extension rate of 10 mm min^−1^.

### Electrical Characterization

Resistivity of the LM‐vitrimer composite was measured using a custom‐built four‐point probe measurement system connected to the source measurement unit (Keithley 2450). The custom four‐point probe measurement system employed a ten pin pogo connector with 1.27 mm spacing between each pin. The thickness of the film was based on the total thickness of the LM‐vitrimer composite film.

### Recycling and Healing of LM‐Vitrimer Composite

The sample used for weight hanging and shape memory was submerged in 5 M sodium hydroxide solution in a capped vial with a magnetic stir bar. The reaction mixture was stirred at 1000 RPM and changes in sample shape and integrity were recorded for approximately four days, that was, until the structural integrity of the sample failed. The liquid metal droplets in solution were collected for future use by filtering the depolymerized mixture through a 200 mesh sieve.

A *ϕ*_*LM*_ = 30% LM‐vitrimer composite was used to demonstrate material healing through Joule heating. A razor blade was used to create a surface cut on the composite. The sample was connected to a power supply and 5 V and 5 A were applied, resulting in material heating. Healing was observed during this process under an optical microscope (Zeiss Axiozoom V.16) and the cut healed in 10 min.

### Reconfigurable Hall‐Effect Sensor Circuit

For creating the pure vitrimer sheet to serve as the circuit base, the uncured vitrimer was cast into a 75 x 60 x 1.5 mm Sylgard 184 mold. This was cured at ambient temperature overnight, followed by curing at 40 °C in a convection oven. The LM‐vitrimer composite was prepared by mixing LM with the uncured vitrimer with an overhead mixer at 200 rpm for 15 min. Then, the composite mixture was allowed to rest at room temperature for 30 min to increase viscosity and then manually mixed to homogenize before screen printing. The composite was then screen printed on the pure vitrimer sheet using a laser cut stencil mask to the specifications of the designed circuit. The printed circuit was cured at ambient temperature overnight, followed by curing at 40 °C in a convection oven. The cured LM‐vitrimer composite interconnects were electrically non‐conductive. These were made conductive through an abrasion force applied on the composite surface. The Hall‐effect sensors (250‐A1326LLHLT‐T, Allegro) and LEDs (Digikey) were attached on to the pre‐defined spaces by using glue. For the demonstration, 2.6 and 0.2 A was applied to the circuit through a power supply and a permanent magnet was brought near the Hall effect sensor to power the LEDs.

## Conflict of Interest

M.J., D.H.H., J.C.W., and M.D.B. are co‐inventors on a provisional patent application relating to this work (US Patent Application No. 63/567/198).

## Author Contributions

J.C.W. and M.D.B. conceived the work and directed the research. All authors designed the experiments. M.J., D.H.H., and R.T. performed and analyzed experiments. All authors contributed to manuscript writing.

## Supporting information

Supporting Information

Supplemental Movie S1

Supplemental Movie S2

Supplemental Movie S3

Supplemental Movie S4

## Data Availability

The data that support the findings of this study are available from the corresponding author upon reasonable request.
