# Supplementary material for: Liquid Metal‐Vitrimer Conductive Composite for Recyclable and Resilient Electronics
Source: Adv Mater. 2025 Jun 1;37(37):2501341. doi: 10.1002/adma.202501341 (PMC12447020; doi:10.1002/adma.202501341)
Supplement: Supplementary file 1 — Supporting Information [file ADMA-37-2501341-s004.pdf]

ADVANCED MATERIALS

Supporting Information

for *Adv. Mater.*, DOI 10.1002/adma.202501341

Liquid Metal-Vitrimer Conductive Composite for Recyclable and Resilient Electronics

Dong Hae Ho, Meng Jiang, Ravi Tutika, Joshua C. Worch and Michael D. Bartlett**

Supplementary Information

Liquid Metal-Vitrimer Conductive Composite for Recyclable and Resilient Electronics

Dong Hae Ho,[1†] Meng Jiang,[2,3†] Ravi Tutika,[1,2] Joshua C. Worch,[2,3*] and Michael D. Bartlett[1,2*]

[1]Mechanical Engineering, Soft Materials and Structures Lab, Virginia Tech, Blacksburg, VA 24061, USA.

[2]Macromolecules Innovation Institute, Virginia Tech, Blacksburg, VA 24061, USA.

[3]Department of Chemistry, Worch Lab, Blacksburg, VA 24061, USA.

[†]These authors contributed equally to this work

[*]To whom correspondence should be addressed: jworch@vt.edu, mbartlett@vt.edu

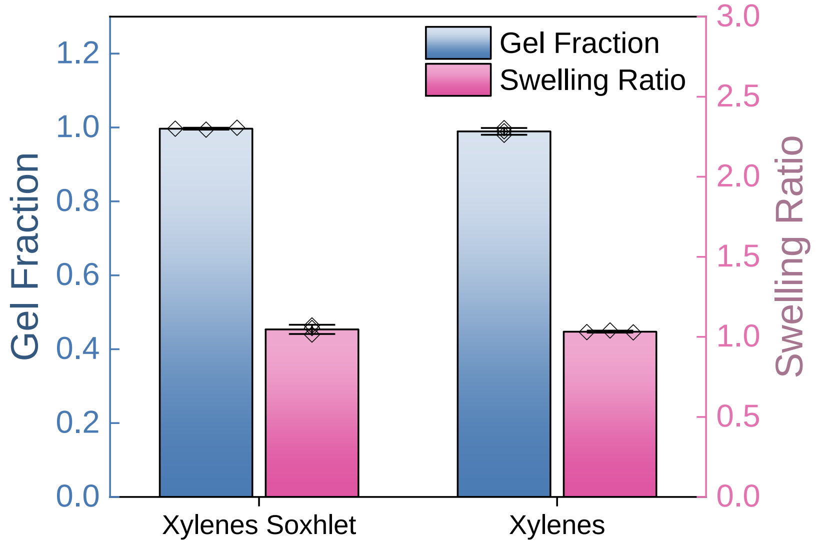

Figure S1. Gel fraction and swelling ratio test for pristine vitrimer at elevated and room temperature in xylenes. Swelling tests were performed in a soxhlet extracter at the boiling point of xylenes (140 °C), which is above the T_g of the vitrimer, and separately at room temperature ($\sim$ 25°C). $n = 3$ from individual samples are presented as scatter symbols and data bars represent mean ± s.d.

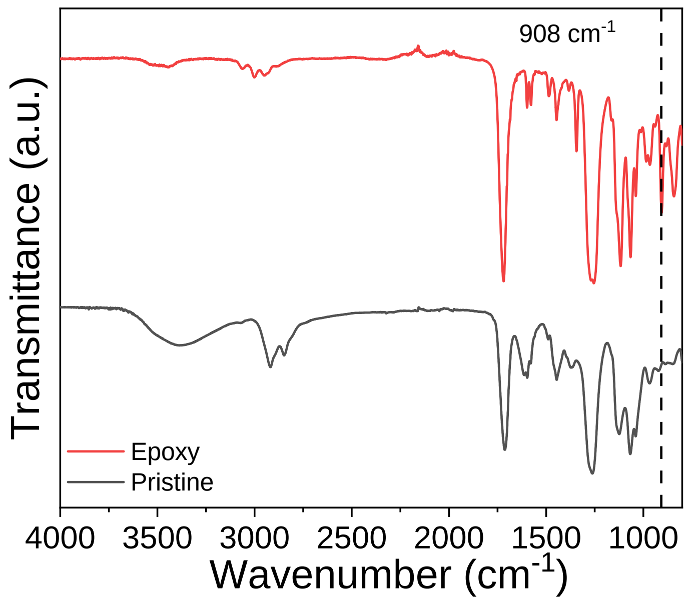

Figure S2. Full range of measured FT-IR spectrum for epoxy resin and pristine vitrimer.

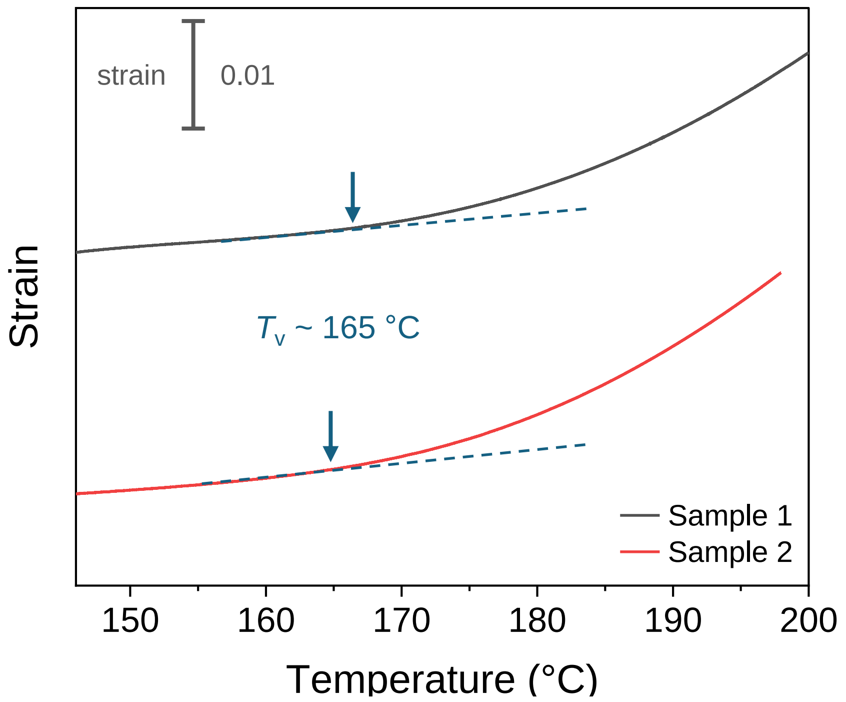

Figure S3. Temperature-dependent thermal expansion of pristine vitrimer. The topology freezing temperature (T_v, $\sim$ 165 °C) is indicated in the graph for two different samples of pristine vitrimer.

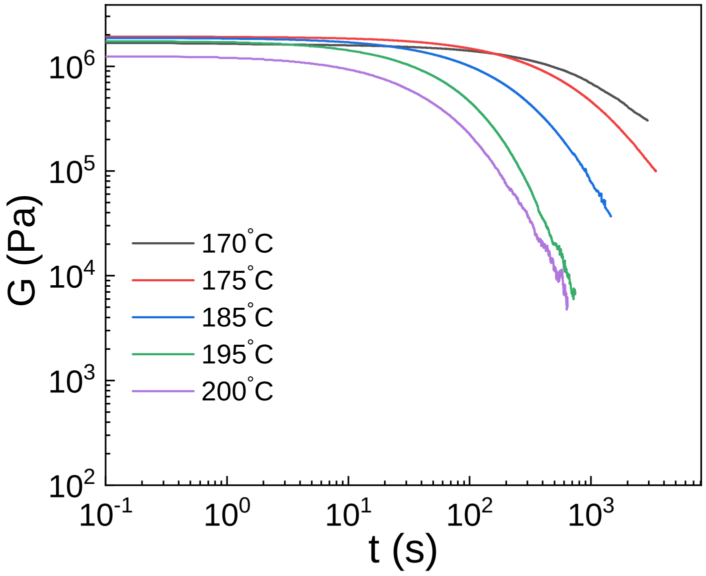

Figure S4. Stress relaxation characterization of the vitrimer on a log-log plot for different temperatures, where G denotes the modulus of the vitrimer.

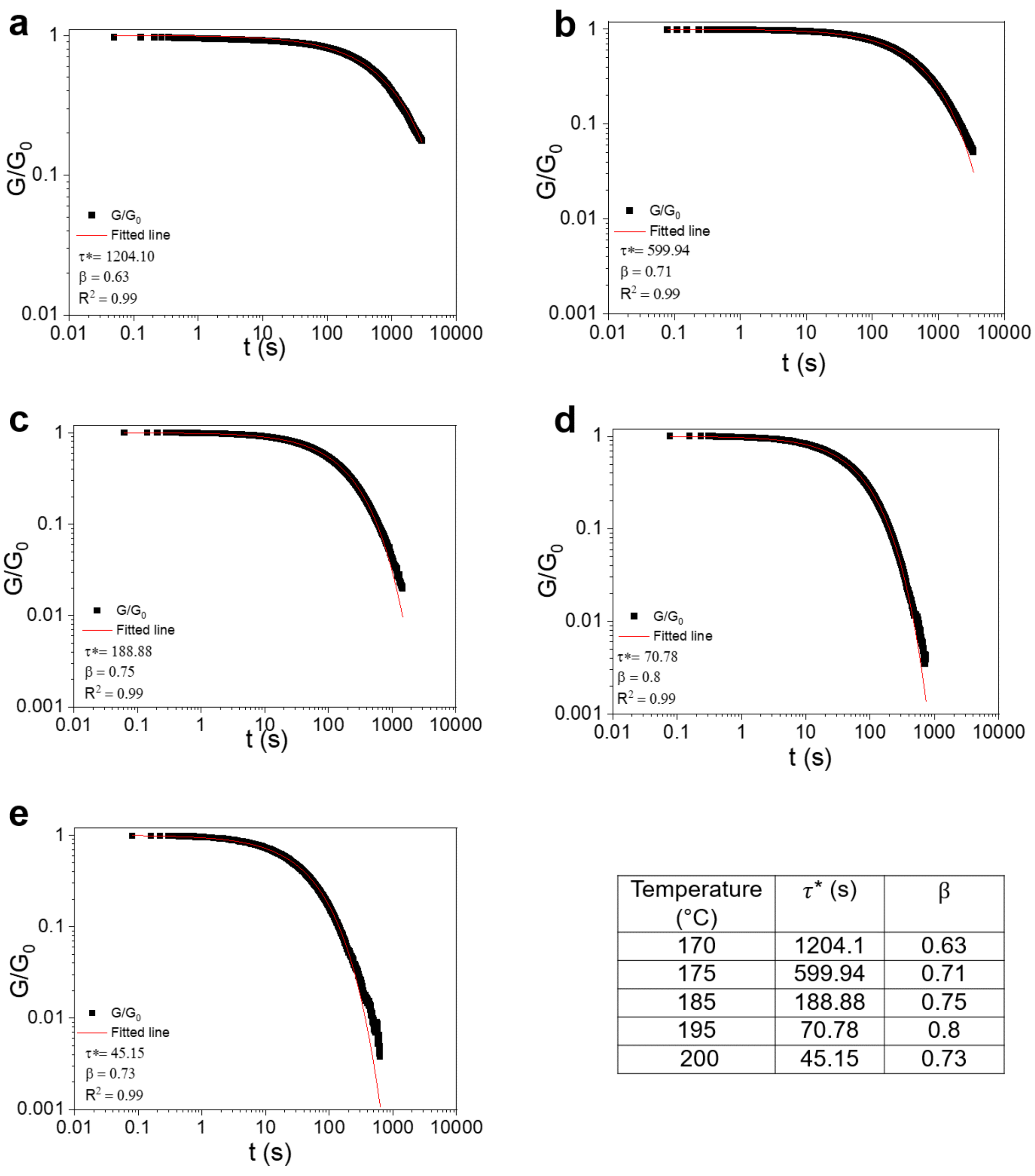

Temperature (°C)	τ^* (s)	β
170	1204.1	0.63
175	599.94	0.71
185	188.88	0.75
195	70.78	0.8
200	45.15	0.73

Figure S5. KWW-model fitting results of the stress relaxation curves and results table. (**a**) 170 °C, (**b**) 175 °C, (**c**) 185 °C, (**d**) 195 °C, and (**e**) 200 °C.

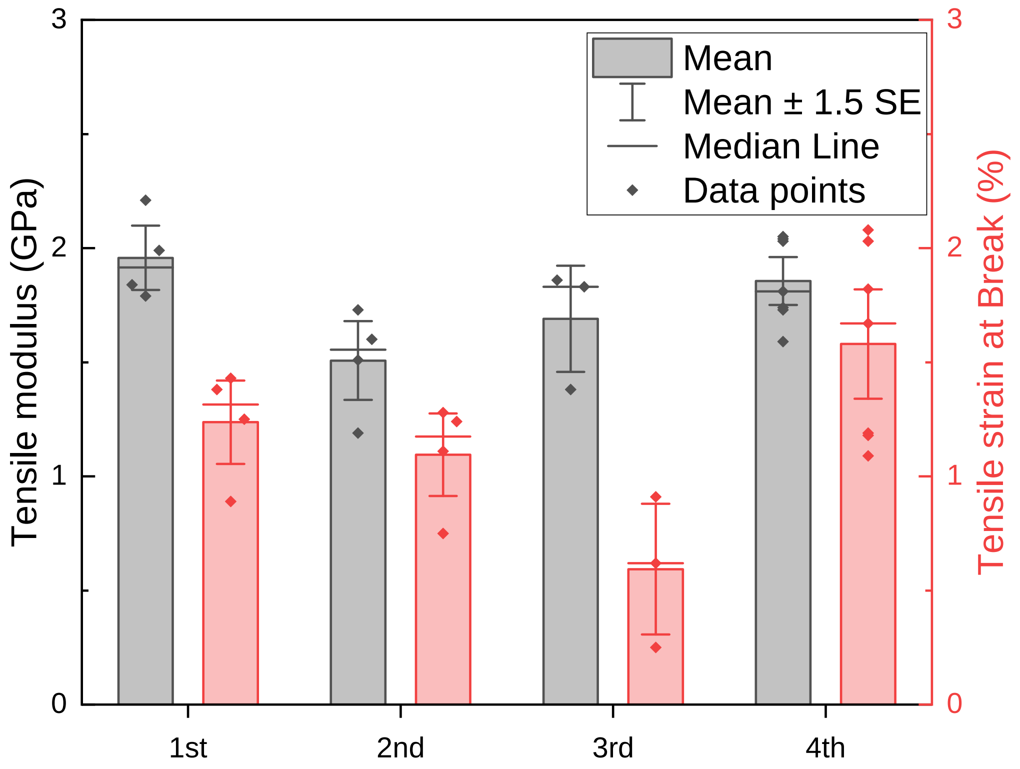

Figure S6. Tensile modulus and strain at break of the vitrimer during 4 cycles of remolding process. Remolding process was performed by pulverizing the vitrimer and applying a vertical force (1.5 t) and heat (170 °C) to the vitrimer powder in a stainless steel mold.

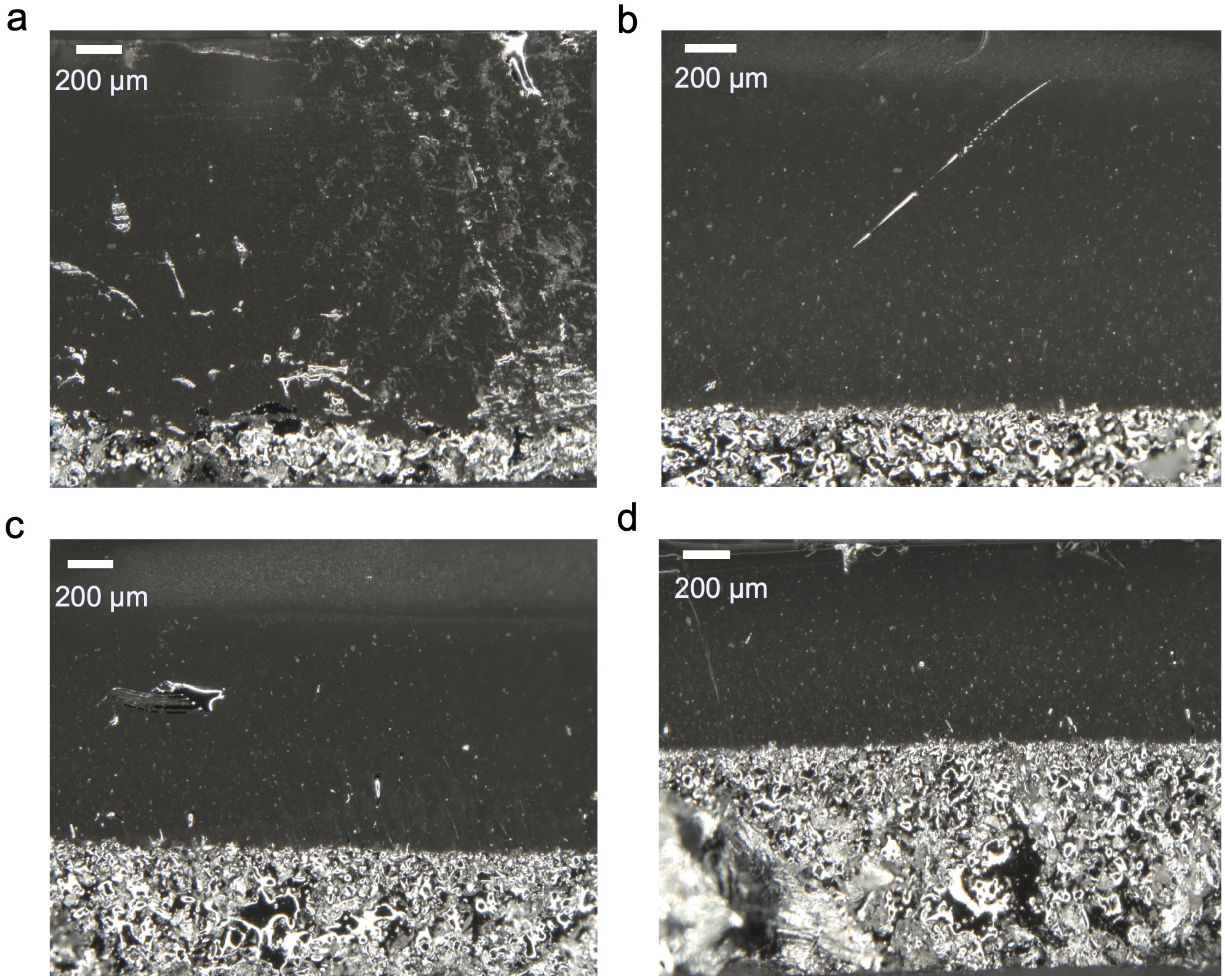

Figure S7. Cross-sectional images of LM-vitrimer composite. (**a**) $\phi_{LM} = 5$ vol%, (**b**) $\phi_{LM} = 10$ vol%, (**c**) $\phi_{LM} = 20$ vol%, (**d**) $\phi_{LM} = 30$ vol%.

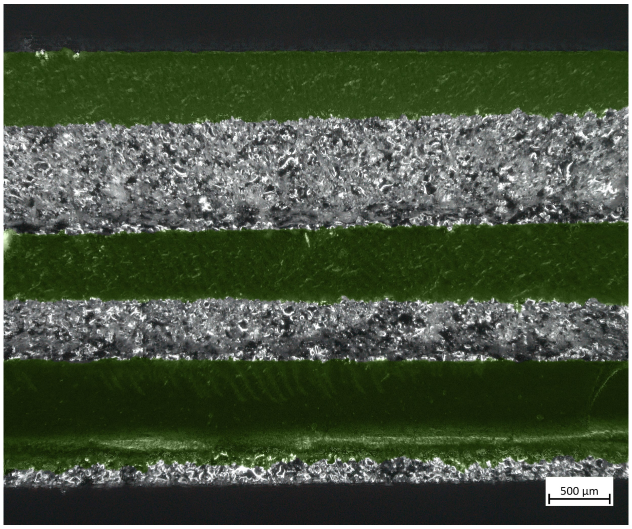

Figure S8. Optical micrograph of a stacked LM-vitrimer composite architecture with A-B A-B A-B (A:LM-vitrimer composite, B:Vitrimer). The bottom layer is $\phi_{LM} = 5\%$, middle layer $\phi_{LM} = 15\%$, and top layer is $\phi_{LM} = 30\%$. Vitrimer regions are pseudo-colored to enhance contrast.

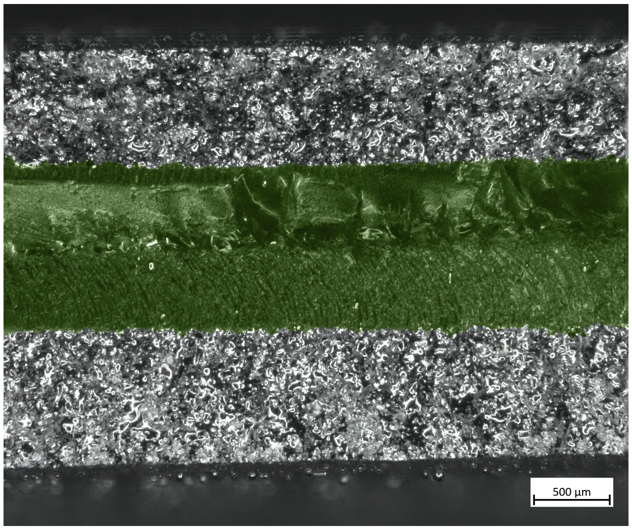

Figure S9. Optical micrograph of a stacked LM-vitrimer composite architecture with A-B B-A (A:LM-vitrimer composite, B:Vitrimer). The two layers of LM-vitrimer composite are $\phi_{LM} = 30\%$. The layers are stacked with LM-composite on both faces with the vitrimer in the middle. Vitrimer regions are pseudo-colored to enhance contrast.

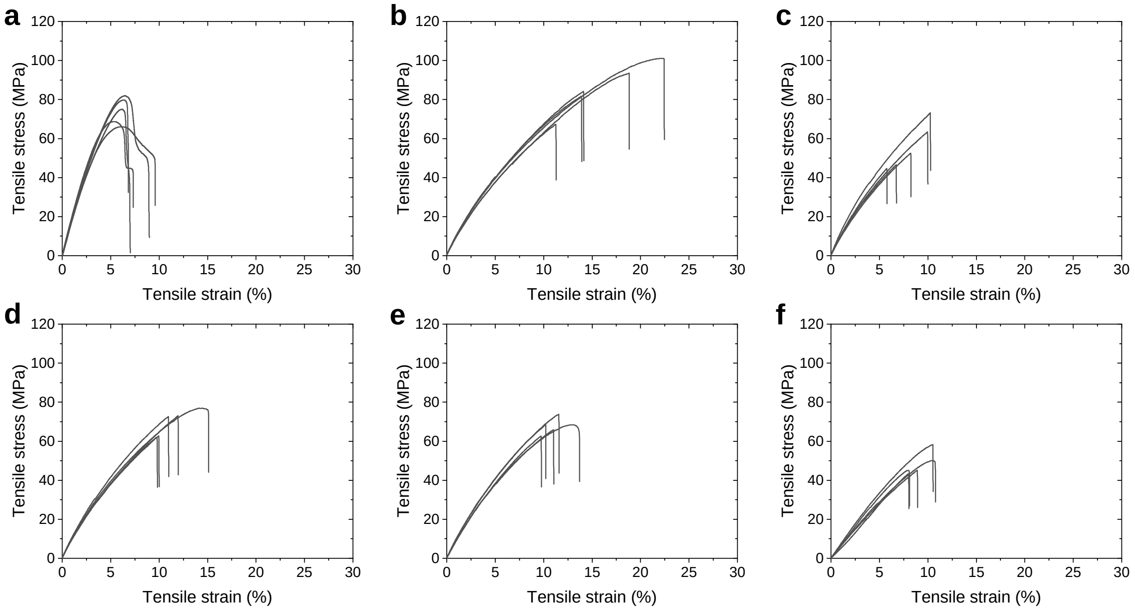

Figure S10. Individual stress-strain curves for every sample used for mechanical characterization. (**a**) $\phi_{LM} = 0\%$, (**b**) $\phi_{LM} = 5\%$, (**c**) $\phi_{LM} = 10\%$, (**d**) $\phi_{LM} = 15\%$, (**e**) $\phi_{LM} = 20\%$, and (**f**) $\phi_{LM} = 30\%$. $n = 5$ for each composition.

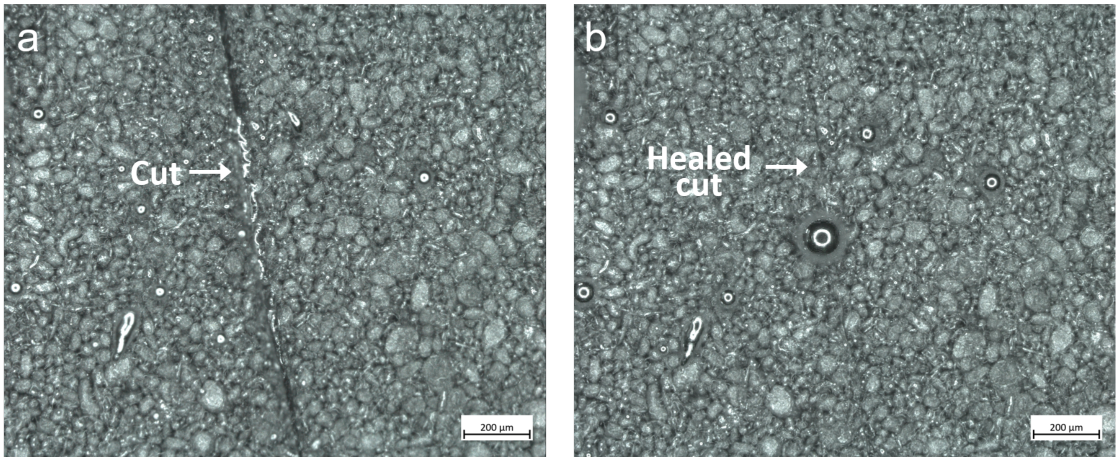

Figure S11. Thermally triggered material healing of LM-vitrimer composites. Optical microscopy image of (**a**) A cut made on the surface of a LM-vitrimer composite sample with $\phi_{LM} = 30\%$. (**b**) Healed cut. A power supply was attached to the sample and Joule heating effect was generated by applying a voltage of 5 V and a current of 5 A to the sample causing the cut to heal in 10 minutes.

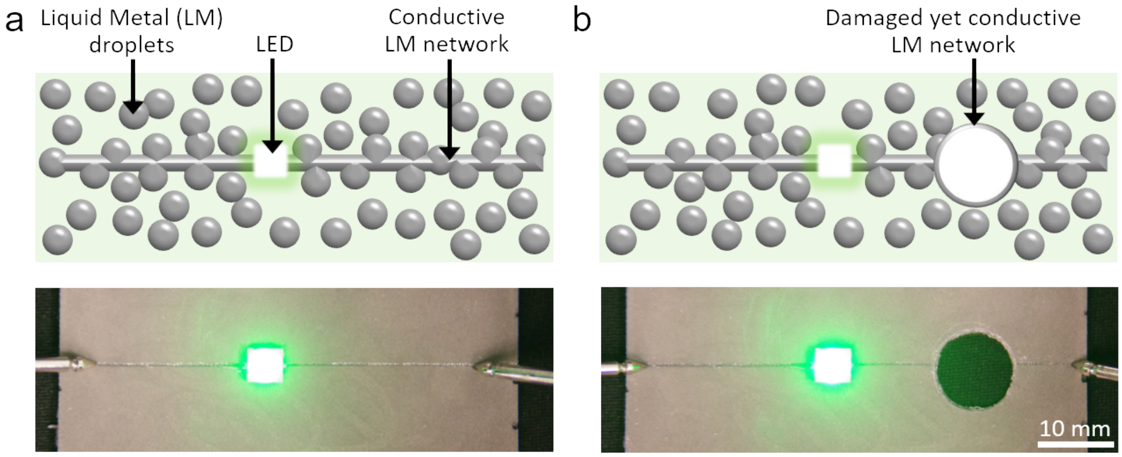

Figure S12. Electrical self-healing of LM-vitrimer composites. (**a**) Schematic and image of an LED circuit created with a LM conductive network made on a LM-vitrimer composite sample with $\phi_{LM} = 30\%$. (**b**) A hole punched in the conductive network removing a section of the composite material maintains the electrical connection indicated by the powered LED. Note that schematics are not to scale.

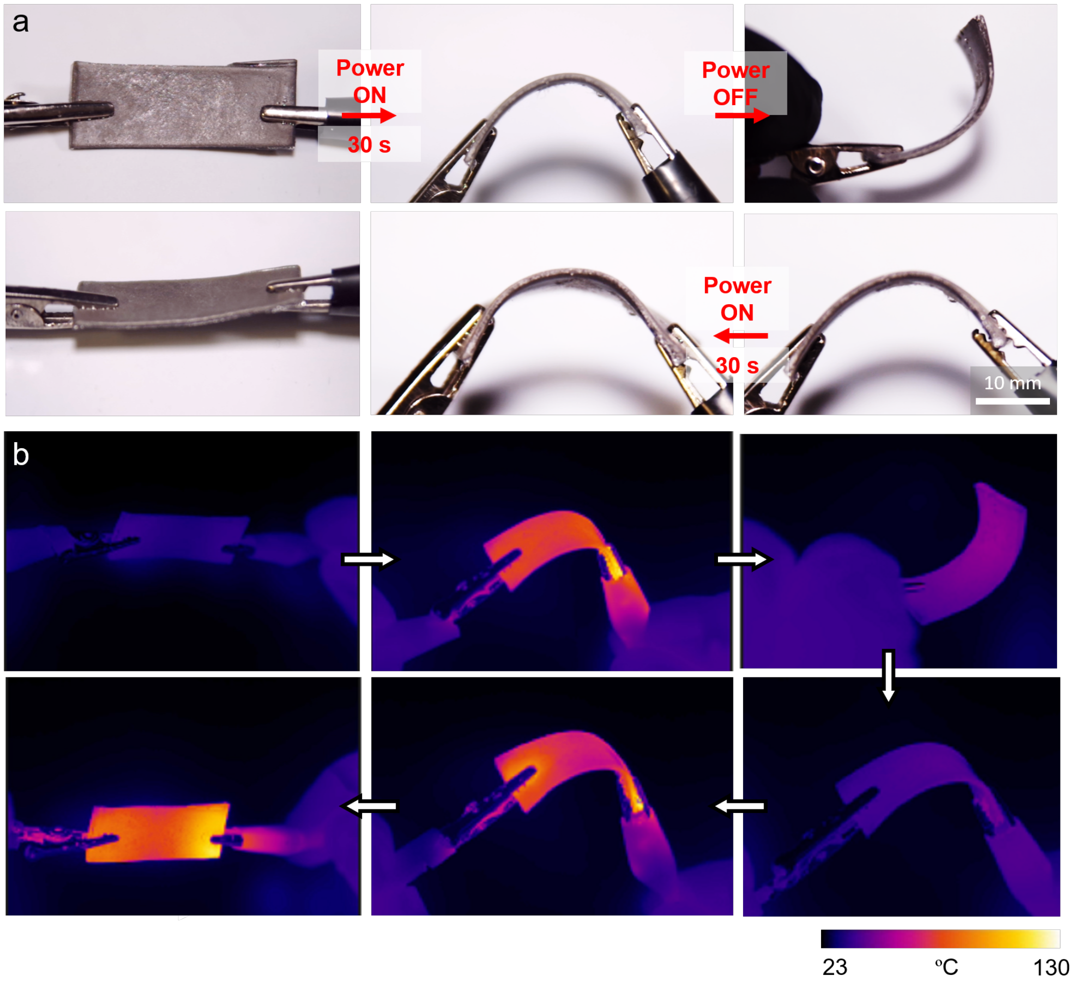

Figure S13. Joule heating demonstration of a LM-vitrimer composite. (**a**) Images of the composite material with $\phi_{LM} = 30\%$ loading when attached to a power supply. When the power supply was turned on, a voltage of 5 V and a current of 5 A was applied. (**b**) Corresponding IR images of the LM-vitrimer composite as it is being reshaped through Joule heating.
